# Crosstalk of nervous and immune systems in pancreatic cancer

**DOI:** 10.3389/fcell.2023.1309738

**Published:** 2023-11-30

**Authors:** Fei-Fei Huang, Wen-Hui Cui, Lan-Yue Ma, Qi Chen, Yang Liu

**Affiliations:** ^1^ The Innovation Centre of Ministry of Education for Development and Diseases, School of Medicine, South China University of Technology, Guangzhou, China; ^2^ Center for Cell Lineage and Development, CAS Key Laboratory of Regenerative Biology, Guangdong Provincial Key Laboratory of Stem Cell and Regenerative Medicine, GIBH-HKU Guangdong-Hong Kong Stem Cell and Regenerative Medicine Research Centre, GIBH-CUHK Joint Research Laboratory on Stem Cell and Regenerative Medicine, Guangzhou Institutes of Biomedicine and Health, Chinese Academy of Sciences, Guangzhou, China; ^3^ China-New Zealand Joint Laboratory on Biomedicine and Health, Guangzhou, China; ^4^ University of Chinese Academy of Sciences, Beijing, China

**Keywords:** pancreatic cancer, peripheral nerve, immune cells, neural-immune crosstalk, PDAC (pancreatic ductal adenocarcinoma)

## Abstract

Pancreatic cancer is a highly malignant tumor known for its extremely low survival rate. The combination of genetic disorders within pancreatic cells and the tumor microenvironment contributes to the emergence and progression of this devastating disease. Extensive research has shed light on the nature of the microenvironmental cells surrounding the pancreatic cancer, including peripheral nerves and immune cells. Peripheral nerves release neuropeptides that directly target pancreatic cancer cells in a paracrine manner, while immune cells play a crucial role in eliminating cancer cells that have not evaded the immune response. Recent studies have revealed the intricate interplay between the nervous and immune systems in homeostatic condition as well as in cancer development. In this review, we aim to summarize the function of nerves in pancreatic cancer, emphasizing the significance to investigate the neural-immune crosstalk during the advancement of this malignant cancer.

## 1 Introduction

Pancreatic ductal adenocarcinoma (PDAC) is the most common type of pancreatic cancer, originating from the cells lining the pancreatic ducts. PDAC is characterized by its highly invasive feature and rapid expansion ability. Despite rapid advancements in medical biotechnology, the incidence of PDAC continues to rise, and its prognosis remains bleak, with 12% of 5-year survival rate (Siegel et al., 2023). Therefore, PDAC has become one of the leading contributors to increased cancer-related deaths worldwide (Siegel et al., 2022). There are multiple risk factors associated with PDAC, including smoking, alcohol consumption, obesity, aging, and family history (Arnold et al., 2009; Genkinger et al., 2009; Permuth-Wey and Egan, 2009; Bosetti et al., 2012). Unfortunately, there are currently no clearly defined effective preventive measures available for PDAC.

The development of PDAC involves several complicated steps that are related with acinar and ductal cells. Acinar cells are responsible for the production and secretion of digestive enzymes, while ductal cells transport these enzymes produced by acinar cells to the intestines. PDAC is induced by pathological changes to the ductal epithelial cells, as well as acinar cells via acinar-to-ductal metaplasia (ADM), which is supported by lineage tracing animal experiments and *in vitro* studies (Rooman and Real, 2012). ADM is a reversible process in which pancreatic acinar cells are transdifferentiated to ductal cells in response to inflammatory signaling, over-activation of KRAS and metabolic stress (Parte et al., 2022). However, ADM becomes irreversible when the oncogene KRAS is persistently overexpressed or abnormal growth factors are persistently produced in pancreas (Storz, 2017). These cells could undergo further deleterious differentiation via a process named pancreatic intraepithelial neoplasia (PanIN), which is a heterogeneous proliferation of tiny flat or columnar epithelial hyperplasia (Cornish and Hruban, 2011). PanIN is considered to be precancerous lesions of pancreatic cancer (Opitz et al., 2021). Therefore, both ADM and PanIN are essential steps in the emergence of PDAC (Giroux and Rustgi, 2017).

The pancreas has a dense neural network that provide a microenvironment to interact with tumor cells by promoting tumor growth or facilitating metastasis (Gasparini et al., 2019). It remains unclear whether pancreatic nerve influence ADM but sensory neurons can directly promote PanIN proliferation through SP-NK-1R signaling and activation of Stat3 (Sinha et al., 2017). Nerves also influence the transformation of PanIN into PDAC by regulating the generation and maintenance of the pancreatic inflammatory response. At the PanIN stage, sprouting of sensory fibers and an increase in neurotrophic factors have been detected, which is associated with an increase of pancreatic inflammatory markers, a process known as neurogenic inflammation (Vera-Portocarrero and Westlund, 2005). This demonstrates that bidirectional signaling between the pancreas and sensory neurons was already established prior to tumor formation (Schwartz et al., 1985; Saloman et al., 2016). After emergence of PDAC, nerves enhance tumor invasion through perineural infiltration to facilitate metastasis and therefore spread of PDAC into the whole body (Chen et al., 2019).

In recent years, there has been a growing knowledge of the intricate interaction between the nervous and immune systems during homeostatic condition, as well as their collaboration in host reactions (Andersson and Tracey, 2012; Chiu et al., 2012). These interactions also play a role in regulating tumor immune evasion and anti-tumor immune responses. Several studies have revealed the existence of complex interactions between the nervous and immune systems within the tumor microenvironment of PDAC, which have significant implications for the development and metastasis of PDAC (Kuol et al., 2018; Cortese et al., 2020). Gaining a deeper understanding of these intricate interactions will provide valuable insights into the mechanisms underlying PDAC progression and facilitate the development of more treatment strategies. Targeting the neuro-immune crosstalk may prove to be an effective approach in PDAC therapy and might also open up new avenues for the treatment of other types of cancer.

This review provides an overview of the role of neuro-immune crosstalk in PDAC, including the influence of neuron on the immune system and *vice versa*. We also discuss current therapeutic approaches aimed at modulating neuro-immune interactions, which may provide valuable insights for future PDAC treatment.

## 2 Neural regulation of PDAC

PDAC, an aggressive tumor with a poor prognosis, affects the nervous system even in its precursor stage known as pancreatic intraepithelial neoplasia (PanIN) (Saloman et al., 2016). The tumor microenvironment refers to the complex cell environment surroundings tumor cells, including immune cells, nerves, endothelial cells, stromal cells, and cancer-associated fibroblasts (Li et al., 2007). The tumor microenvironment plays a significant role in tumor growth, invasion, metastasis, and treatment response (Hanahan and Coussens, 2012; Yuan et al., 2016). The nervous system is an essential component of the tumor microenvironment that contributes to tumor initiation and progression (Shurin et al., 2020).

The pancreas contains a dense network of nerves, with the head (proximal duodenal lobe) having a significantly higher density of nerve plexus (Berthoud and Powley, 1991; Fasanella et al., 2008). The innervation of the pancreas involves nerves through spinal cord and the vagus nerve, both conveying sensory information, as well as the parasympathetic and sympathetic nerves (Lindsay et al., 2005; Teff, 2008).

### 2.1 Perineural invasion

Perineural invasion (PNI) occurs when tumor cells infiltrate along nerves or within the outer sheath and spaces surrounding nerves (Bockman et al., 1994; Yang et al., 2020a). Perineural invasion is a notable characteristic of PDAC, present in over 80% of PDAC patients, even at early stages like pancreatic intraepithelial neoplasia (Ceyhan et al., 2009; Saloman et al., 2016). Perineural invasion is linked to the neural innervation of PDAC and correlates with pain experienced by these patients (Ceyhan et al., 2009; Bapat et al., 2011). Additionally, perineural invasion provides a pathway for tumor invasion into nearby tissues and enhances the local invasive capacity of the tumor (Ceyhan et al., 2009). The extent of perineural invasion in PDAC is significantly associated with postoperative prognosis, making it a predictive factor for survival. Controlling perineural invasion is therefore crucial in PDAC treatment.

Neurotrophic factors secreted by the nervous system play a critical role in PDAC, influencing the perineural invasion process through autocrine or paracrine mechanisms (Huang et al., 2018). Schwann cells and macrophages within the nerves can regulate tumor cell behavior and contribute to PNI exacerbation (Demir et al., 2014; Bakst et al., 2017). Furthermore, specific axon guidance molecules, such as Semaphorin 3D, Plexin D1 and serine, have been found to promote PNI in PDAC (Jurcak et al., 2019; Banh et al., 2020; White and Wang, 2021).

### 2.2 Sympathetic and parasympathetic nerves in PDAC

**FIGURE 1:**
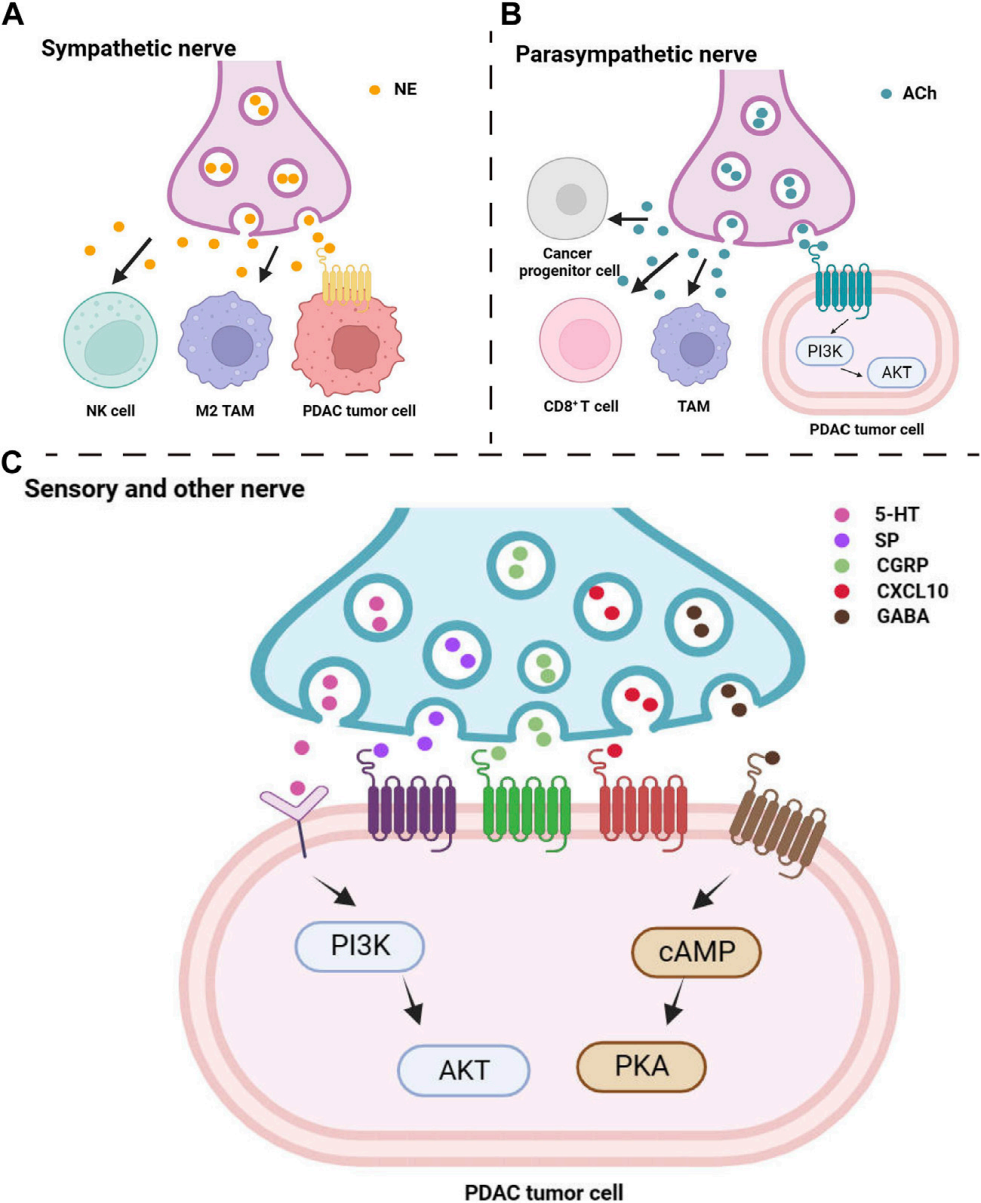
Peripheral nerve release neuropeptide to influence pancreatic tumor cells and microenvironmental immune cells. Multiple neuropeptides from sympathetic nerve **(A)**, parasympathetic nerve **(B)**, sensory and other nerve **(C)** directly target to tumor cells or influence cancer progress by indirectly target to immune cells.

An increase in pancreatic neural plexus density is associated with PDAC development (Bockman et al., 1994). The sympathetic nervous system has a dual role in PDAC. On one hand, it has been reported that the sympathetic nervous system can directly influence the growth of PDAC through beta-adrenergic signaling by releasing norepinephrine (Guo et al., 2013; Kim-Fuchs et al., 2014) (Figure 1A). On the other hand, some evidence suggests that the sympathetic nervous system exhibits inhibitory effects on pancreatic cancer, either directly (Guillot et al., 2022) or indirectly via NK cells (Song et al., 2017).

Research has indicated that parasympathetic nerve signaling can slow down the progression of pancreatic tumors mediated by inflammation (De Couck et al., 2016). Parasympathetic nerve blockage promotes the growth of PDAC and shortens overall survival (Partecke et al., 2017), while the use of muscarinic agonists inhibits the occurrence of PDAC and prolongs overall survival (Renz et al., 2018a) (Figure 1B). However, besides its involvement in PDAC development through suppressing inflammatory responses, promoting immune regulation, and anti-tumor immune reactions, activation of the parasympathetic nervous system is also believed to be associated with poor prognosis in PDAC (Zhang et al., 2016a; Zhang et al., 2016b).

Thus, the roles of sympathetic and parasympathetic nerves in PDAC are complex and sometimes controversial, requiring further investigation.

### 2.3 Sensory nerve in PDAC

Severe and persistent pain is a common accompanying symptom of PDAC and is frequently associated with the prognosis of patients in advanced stages of the disease (Laitinen et al., 2017). PDAC pain is a complex process involving various mechanisms such as local tumor infiltration, nerve compression, and inflammation (Mantyh et al., 2002; Wang et al., 2021).

While the mechanisms of PDAC pain are not yet fully understood, it is well established that neurogenic inflammation plays a significant role to induce pain (Wang et al., 2021). Neurogenic inflammation is an inflammatory response that influences the tumor microenvironment, involving vascular dilation and plasma protein extravasation due to peripheral release of substance P and calcitonin gene-related peptide, both of which are neuropeptides (Schmelz and Petersen, 2001) (Figure 1C). Pain-associated sensory neurons primarily release substance P and are involved in the transmission of pain signals (Pernow, 1953). Calcitonin gene-related peptide, belonging to the calcitonin peptide family, is mainly synthesized and secreted by sensory neurons within peripheral tissues. Studies have shown that calcitonin gene-related peptide usually co-localizes with substance P and both play a role in the transmission of sensory signals, particularly pain (Gibbins et al., 1985).

Therefore, neurogenic inflammation results in persistent neuropathic pain, which greatly influences the patient’s quality of life (Breivik et al., 2006; Grace et al., 2014). Tumor cells not only mechanically affect sensory nerve, they also release inflammatory factors that stimulate nerve endings and cause pain (Mantyh et al., 2002). Sensory neurons can also heighten nociceptive hypersensitivity by releasing specific proteins like substance P and calcitonin gene-related peptide. Additionally, sensory neurons can secrete chemokines that act on chemokine receptors, which enhances the sensitivity of nociceptive neurons and promoting neurogenic inflammation (White et al., 2009). Hirth et al. discovered a potential link between chemokines CXCL10 and CCL21 with PDAC pain, and pain relief can be achieved by neutralizing these chemokines (Hirth et al., 2020).

### 2.4 GABAergic and serotonergic neurons in PDAC

In addition to substance P, calcitonin gene-related peptide and chemokines, neurotransmitters play a crucial role in promoting the development and survival of PDAC cells, including Gamma-aminobutyric acid (GABA) and 5-hydroxytryptamine (5-HT), commonly known as serotonin (Figure 1C).

Suppression of the inhibitory neurotransmitter GABA has been reported to enhance the invasion and growth of tumor cells (Al-Wadei and Schuller, 2009; Banerjee et al., 2016). Studies have shown that supplementing with GABA can decrease cAMP levels and inhibit the release of the pro-inflammatory cytokine interleukin-6 (IL-6), thus preventing pancreatic inflammation from progressing to PDAC. GABA supplementation can also inhibit the abnormal signaling pathway caused by ethanol-induced inhibition of cAMP-mediated PKA signaling, suggesting it as a promising preventive approach for pancreatitis-related PDAC and PDAC caused by long-term alcohol consumption (Al-Wadei et al., 2013; Banerjee et al., 2016).

It is well-known that tumor cells primarily rely on glycolysis as their metabolic method under both aerobic and anaerobic conditions (Hsu and Sabatini, 2008). This phenomenon, known as the Warburg effect, is closely associated with the malignant progression of PDAC (Yang et al., 2020b). Research indicates that serotonin can modulate the Warburg effect by activating the PI_3_K/Akt/mTOR pathway, thereby enhancing the survival capabilities of tumor cells (Jiang et al., 2017). Lyn, a kinase belonging to the Src family, has been found to play a promoting role in the serotonin-induced PI_3_K/Akt/mTOR pathway. Knocking down Lyn leads to significant reductions in both serotonin levels and the extent of the Warburg effect (Jiang et al., 2017).

Furthermore, HTR2B, a vital subtype of the serotonin receptor (Nebigil et al., 2001), has a positive relationship with serotonin expression and can facilitate the development of PDAC (Jiang et al., 2017). Controlling the activity of HTR2B through knockdown methods may contribute to reduce disease progression and increase overall survival in PDAC patients (Jiang et al., 2017). The expression of HTR2B can independently predict the invasiveness of PDAC, suggesting that it could be a potential target for PDAC therapy. Lowering down the levels of serotonin through different approaches, such as targeting key factors involved in its production, shows potential in slowing down the progression of PDAC.

To sum up, there is a correlation between changes in the density of the pancreatic neural plexus and the development of PDAC (Ceyhan et al., 2009). However, more research is necessary to investigate the complex interaction of various neural networks in PDAC, in order to gain a better understanding of neuronal regulation of PDAC to intervene disease progression.

## 3 The influence of nerve to immune cells in pancreatic cancer

**FIGURE 2:**
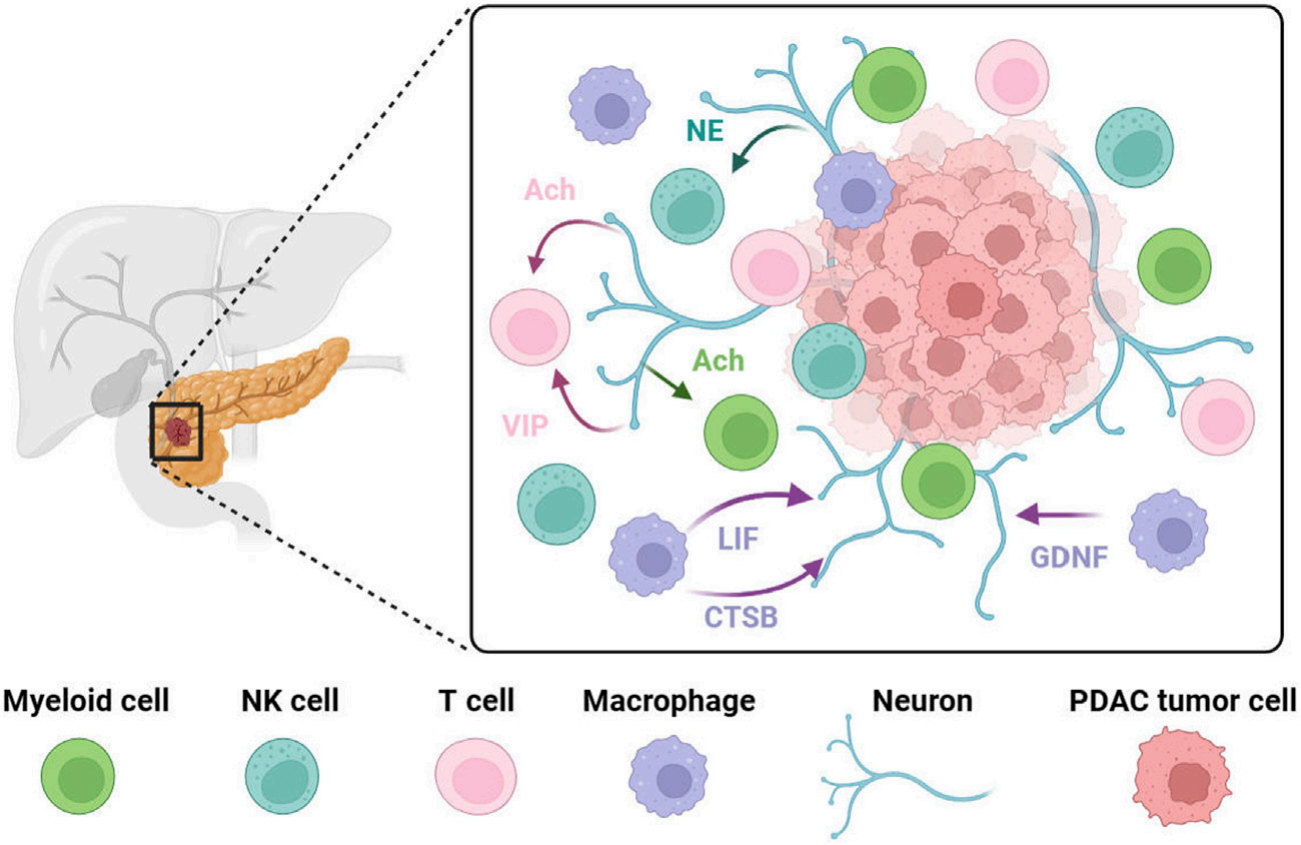
PDAC progression is associated with crosstalk between neurons and immune cells. Pancreatic nerves release VIP or acetylcholine to target T cells to reduce their anti-tumor effect or to inhibit interferon production from T cells. Norepinephrine has been shown to inhibit NK cell activity via the β-adrenergic receptor *in vitro*. Vagus nerve-derived acetylcholine exerts its effect on myeloid cells, inhibiting the release of pro-inflammatory cytokines. In PDAC, tumor-associated macrophages at the tumor invasion site secrete high levels of GDNF. These recruited tumor-associated macrophages promote perineural invasion by secreting large amounts of cathepsin B (CTSB) to degrades collagen IV facilitating the invasion and spread of tumor cells along the nerve. LIF has a positive correlation with PDAC-associated neural remodeling. NE, norepinephrine; Ach, acetylcholine; VIP, vasoactive intestinal peptide; LIF, leukemia inhibitory factor; CTSB, cathepsin B; GDNF, Glial-derived neurotrophic factor.

In PDAC, both the nervous system and the immune system play important roles, respectively, in influencing tumorigenesis, progression, treatment, and prognosis. However, whether there is an interaction between the two systems and how such an interaction takes place is still under investigation. This part will focus on the crosstalk between the nervous system and the immune system in PDAC (Figure 2).

### 3.1 The sympathetic system

Sympathetic nerves are predominantly believed to promote tumor development, as seen in prostate and breast cancer (Bauman and McVary, 2013; Kamiya et al., 2019). This promotion may be attributed to the production of nerve growth factor (NGF) (Renz et al., 2018b) and potentially angiogenesis (Kim-Fuchs et al., 2014). In the case of PDAC, however, apart from the existing studies on sympathetic promotion of tumor growth, there have also been reports of sympathetic restriction of PDAC growth. This cancer-protective effect is primarily achieved by modifying the tumor microenvironment in PDAC. It has been demonstrated that PDAC tumor cells tended to grow after both surgical sympathectomy and peripheral chemical sympathectomy achieved by using the neurotoxin 6-hydroxydopamine (6-OHDA), respectively. This effect is likely caused by an increase in CD163 macrophage (M_2_-type macrophage) cells (Guillot et al., 2022), which have the potential to drive tumor progression by suppressing T cell-mediated anti-tumor immunity (Etzerodt et al., 2012; Cheng et al., 2017).

Natural killer (NK) cells play a crucial role in tumor recognition and tumor cell elimination (Vivier et al., 2012; Chester et al., 2015). It has been demonstrated in a mouse model of PDAC that anti-tumor immunity was suppressed through peripheral chemical sympathectomy. This suppression was observed by inhibiting the expression of NKG2D and CCR5 in NK cells (Song et al., 2017). However, contradictory findings have been reported *vitro* studies, where norepinephrine has been shown to inhibit NK cell activity via the β-adrenergic receptor (Ben-Eliyahu et al., 2000). This suggests that sympathetic nerves may have a dual effect on NK cells. Although these pioneer works investigated the role of sympathetic nerves on NK cells in PDAC, further evidence is necessary to fully understand their relationship as microenvironment of PDAC.

To sum up, sympathetic nerves exhibit distinct mechanisms in different components of the PDAC environment and have demonstrated both pro- and anti-tumor effects in various studies. These differences may also be associated with α and β receptors, with β receptors consistently linked to tumor promotion in existing literature (Renz et al., 2018b). Conversely, the role of α receptors remains unknown and requires further investigation in subsequent studies.

### 3.2 The parasympathetic system

The vagus nerve is the primary component of the parasympathetic nerves in pancreas and is involved in the production of inflammatory factors as well as the regulation of the immune systems. This is mainly achieved through the binding of the neurotransmitter acetylcholine to muscarinic-type receptors (M-receptors) or nicotinic-type receptors (N-receptors) (Borovikova et al., 2000; Huston et al., 2009). Numerous studies have demonstrated that the vagus nerve plays a protective role in various tumors (Gidron et al., 2005; Reijmen et al., 2018; Li et al., 2023). For instance, in pancreatic cancer, vagotomy, which involves the cutting of the vagus nerve, can lead to increased tumor growth, worsened survival, and higher levels of tumor-associated macrophages and TNF-α (Partecke et al., 2017). Further investigation has revealed that muscarinic receptors can inhibit tumorigenesis by down-regulating MAPK and PI_3_K/AKT signaling through CHRM1 receptors in tumor cells. Additionally, activating muscarinic receptors has been found to increase levels of circulating TNFα and CD11b^+^ myeloid cells (Renz et al., 2018a). Nicotinic receptors also contribute to vagus nerve-mediated tumor suppression. Tracy’s study describes the vagus nerve’s role in suppressing inflammation as a “cholinergic anti-inflammatory pathway,” (Tracey, 2002) which relies on the nAChR (Wang et al., 2003). This pathway explains how the vagus nerve may reduce the production of inflammatory cytokines in PDAC. Acetylcholine released by the vagus nerve exerts its effect through the α7 nicotinic acetylcholine receptor (α7nAChR) found on macrophages, inhibiting the production and release of pro-inflammatory cytokines like TNF, IL-1β, IL-6, and IL-18 (Borovikova et al., 2000; Wang et al., 2003; Falvey, 2022).

There are clinical studies that suggest the vagus nerve can reduce the risk of death in metastatic PDAC (De Couck et al., 2016). Both muscarinic and nicotinic receptors play a role in inhibiting PDAC. Research has demonstrated that subdiaphragmatic vagotomy, the cutting of the vagus nerve below the diaphragm, leads to accelerated PDAC development. Normal cellular phenotypes can be restored by using the muscarinic agonist bethanechol. However, similar to sympathetic nerves, the vagus nerve also plays a dual role in PDAC by modulating the tumor microenvironment.

Research has indicated that when perineural invasion occurs in PDAC, there is an elevated level of acetylcholine, which promotes tumor growth primarily by affecting T cell responses. Acetylcholine impairs PDAC cells’ ability to recruit CD8^+^ T cells and reduces their anti-tumor immunity by inhibiting CCL5 through HDAC1-mediated mechanisms. Additionally, acetylcholine directly inhibits interferon production by CD8^+^ T cells in a dose-dependent manner through the nicotinic acetylcholine receptor (nAChR). This promotes Th2 polarization of T cells and a decrease in the Th1/Th2 ratio, which is essential balance in immune response, thereby facilitating tumor growth and lowering the survival rate (Yang et al., 2020a).

These existing studies suggest that the exact functions of acetylcholine are complex and sometimes controversial, which requires further investigation taking into account the specific context of acetylcholine receptors, components of the tumor microenvironment as well as the progression stages of the disease.

### 3.3 Other neuromodulators

#### 3.3.1 Vasoactive intestinal peptide (VIP)

Vasoactive intestinal peptide is a neuropeptide consisting of 28 amino acids. It is primarily released by neurons and immune cells, with a crucial role as a neurotransmitter in immune regulation and vasodilation (Delgado and Ganea, 2013). VIP’s involvement in tumor growth and metastasis is linked to its regulation by immune cells (Moody et al., 1993; Fernandez-Martinez et al., 2009). There is a high expression of VIP receptors on immune cells, and in the case of PDAC, these receptors are upregulated during T cell activation. Tumor cells produce VIP, which acts on T cells through the paracrine manner, inhibiting their anti-tumor activity and promoting the development of Treg and Th2 cells. Inhibiting VIP receptors using VIP-R antagonists has shown promise in inhibiting PDAC progression and is considered to be a viable treatment option for the condition (Ravindranathan et al., 2022).

#### 3.3.2 Netrin G1

Netrin G1 is a neuronal cell adhesion molecule that belongs to the netrin family. Its primary function is to serve as a long-range chemical axon guidance cue during development (Zhang et al., 2016c). In human PDAC tissue, Netrin G1 is found to be overexpressed compared to normal tissue, and its expression is negatively correlated with survival rates. Both *in vivo* and *in vitro* studies have revealed that treatment with anti- Netrin G1 monoclonal antibodies effectively inhibits tumor formation, indicating that Netrin G1 may be a potential target for PDAC therapy. Knockdown of Netrin G1 in tumor-associated fibroblasts in PDAC has been shown to reduce the presence of immuno-suppressive factors (Francescone et al., 2021). However, it is important to note that the expression of IL15, a crucial factor in activating NK cells and enhancing the anti-tumor activity of CD8^+^ T cells, is significantly higher in tumor cells compared to Netrin G1 (Fehniger et al., 2002; Waldmann, 2003; Klebanoff et al., 2004). This suggests that reducing Netrin G1 expression may activate NK cells, facilitating to create a less immunosuppressive tumor microenvironment and inhibiting PDAC progression. More comprehensive research on Netrin G1 and clinical trials involving anti- Netrin G1 monoclonal antibodies are necessary to fully understand treatment efficacy and potential adverse reactions.

## 4 The influence of immune cells on the nervous system in pancreatic cancer

Despite immune cells constituting nearly 50% of PDAC’s cellular component (Clark et al., 2007), PDAC still exhibits an immunosuppressive tumor microenvironment because of the abundance of immunosuppressive cells over anti-tumor effector cells. This immunosuppressive environment is characterized by T-cell exhaustion and the infiltration of various immunosuppressive cells, including tumor-associated macrophages (TAMs), myeloid-derived suppressor cells (MDSCs), and regulatory T cells (Tregs) (Liu et al., 2019). These immunosuppressive cells contribute to the reduction of anti-tumor immunity and the promotion of tumor growth through the secretion of cytokines and the inhibition of CD8^+^ T cells (Zheng et al., 2013). Additionally, there is a subset of cells that influence the nervous system to promote tumor development, with tumor-associated macrophages being the most extensively studied in this regard.

### 4.1 Origin of tumor-associated macrophages

A significant influx of infiltrating tumor-associated macrophages is observed as early as the PanINs stage of the pancreatic cancer, persisting throughout PDAC (Clark et al., 2007). Initially, monocytes are recruited into the PDAC microenvironment and undergo differentiation into tumor-associated macrophages under the chemotactic influence of various factors secreted by tumor cells, Schwann cells, and other cells, resulting in the formation of a unique tumor microenvironment (Franklin et al., 2014; Bakst et al., 2017). Tumor cells not only secrete high levels of colony-stimulating factor 1 (CSF-1) to independently recruit macrophages (Zhu et al., 2014) but also release the chemokine CCL2 in conjunction with Schwann cells, facilitating the infiltration of inflammatory monocytes via the CCL2/CCR2 axis (Sanford et al., 2013; Bakst et al., 2017). Furthermore, the vascular endothelial growth factor (VEGF)/epidermal growth factor receptor (EGFR) signaling axis (Dineen et al., 2008) and the hypoxic tumor microenvironment are also implicated in macrophage recruitment in PDAC (Doedens et al., 2010).

### 4.2 Important signaling mediating crosstalk between nerve and tumor-associated macrophages

#### 4.2.1 Glial-derived neurotrophic factor (GDNF)

Glial-derived neurotrophic factor (GDNF) is a crucial neurotrophic factor that supports the survival of neural cells in both the central and peripheral nervous systems (Airaksinen and Saarma, 2002; Ito et al., 2005). When GDNF initially binds to the GDNF family receptor α1 (GFRα1), this complex then activates the transmembrane proto-oncogene Ret receptor (RET) by inducing phosphorylation of RET tyrosine residues. Therefore, the interaction between GFRα1 and RET is necessary for a response to GDNF.

In PDAC, activated and recruited tumor-associated macrophages at the tumor invasion site secrete high levels of GDNF (Cavel et al., 2012). This leads to phosphorylation of RET and subsequent activation of extracellular signal-regulated kinases (ERK) in PDAC tumor cells, ultimately promoting the perineural invasion of pancreatic cancer cells. Interestingly, dorsal root ganglia can release soluble GFRα1 even in the absence of cancer cell expression of GFRα1. The GFRα1 released by nerves further enhances RET activation and amplifies cancer cell perineural invasion (He et al., 2014).

Notably, the effect of GDNF is more pronounced in cells with the G691S RET polymorphism, which exhibits higher activation of the MAPK signaling pathway. This suggests a direct correlation between the G691S RET single nucleotide polymorphism and the aggressive growth of pancreatic cancers (Okada et al., 1999; Sawai et al., 2005).

#### 4.2.2 Cathepsin B

Cathepsin B (CTSB) is a member of the papain superfamily and plays a role in various physiological processes, including protein degradation, lipid metabolism, and antigen presentation (Mort et al., 1997). In tumors, cathepsin B contributes to tumor cell invasion by regulating angiogenesis, disrupting cellular junctions, and cleaving cell adhesion molecules (Olson and Joyce, 2015). In PDAC, it has been reported that CCL2 derived from Schwann cells facilitates the differentiation of monocytes into tumor-associated macrophages. These recruited tumor-associated macrophages promote perineural invasion by secreting large amounts of cathepsin B. Cathepsin B effectively degrades collagen IV, an essential component of the protective nerve bundle membrane, thus facilitating the invasion of tumor cells into nerves and their spread along the nerve (Bakst et al., 2017).

#### 4.2.3 Leukemia inhibitory factor (LIF)

Prior to the onset of perineural invasion, significant alterations occur in several neural compartments, collectively known as PDAC-associated neural remodeling (PANR) (Demir et al., 2010; Saloman et al., 2016). This process is promoted by the crosstalk between stromal cells and nerves. Co-culturing macrophages with fibroblasts greatly enhances the secretion of leukemia inhibitory factor by fibroblasts. Leukemia inhibitory factor activation subsequently triggers JAK/STAT3/AKT signaling, promoting glial cell differentiation, inducing their migration, and increasing plasticity in dorsal root ganglion neurons by extending the number of neurite protrusions and cytosolic area. The experimental results support a positive correlation between LIF and PDAC-associated neural remodeling, highlighting the potential of serum LIF as a stratification marker for PDAC. Furthermore, the combined detection of LIF and CA19-9, which is an important molecular marker for pancreatic cancer, could be utilized as a diagnostic and predictive marker (Bressy et al., 2018).

#### 4.2.4 Macrophage migration inhibitory factor (MIF) and CD74

Macrophage migration inhibitory factor (MIF) is a protein secreted by various immune and epithelial cells, including macrophages with pro-inflammatory properties (Funamizu et al., 2013). Macrophage migration inhibitory factor promotes tumor development through multiple pathways and its levels are significantly elevated in PDAC (Wang et al., 2018). CD74, a protein on the cell membrane, acts as a surface receptor for macrophage migration inhibitory factor, whose main function is to cooperate with MHCII molecules, regulating antigen presentation and affecting the proliferation and survival of B cells (Cohen and Shachar, 2012). Additionally, CD74 can also participate in signal transduction pathways (Becker-Herman et al., 2005) and is considered a prognostic factor in cancer, as higher expression of CD74 indicates tumor progression. In PDAC, CD74 levels progressively increase with disease progression. Through the PI_3_K/AKT/EGR-1 pathway, CD74 enhances tumor invasion and promotes neuroplasticity by increasing GDNF secretion, thereby facilitating tumor invasion (Zhang et al., 2021). Knocking down CD74 has been found to reduce the invasive ability of PDAC and the growth index of dorsal root ganglia, achieving an inhibitory effect on PNI. Targeting CD74 for PDAC treatment shows promising potential.

## 5 Therapeutic targets of PDAC

The limitations of surgical intervention and the strong resistance of PDAC to chemotherapy enables PDAC treatment to be a challenging task (Andersson et al., 2009; Lambert et al., 2019). Molecular therapy, utilizing specific drugs or substances to target specific molecules or sites within tumor cells, is not universally effective for PDAC patients, particularly those with advanced PDAC (metastatic pancreatic cancer) (Remond et al., 2022). Immunotherapy, an emerging field in cancer treatment, aims to activate the patient’s immune system to inhibit and eliminate cancer cells. However, the immunosuppressive tumor microenvironment of PDAC limits the efficacy of immunotherapies such as immune checkpoint inhibitors (ICI), CAR-T cell therapy, or vaccines in PDAC treatment (Zhang et al., 2022). The interaction between the nervous and immune systems plays a crucial role in tumor growth and proliferation, underscoring its significance in PDAC treatment (Kuol et al., 2018; Cortese et al., 2020). The next section will discuss several PDAC treatment approaches based on neuro-immune crosstalk.

### 5.1 Combination of VIP receptor antagonists and ICI

As mentioned earlier, when VIP binds to VIP receptors on T cells, it sends inhibitory signals that suppress the anti-tumor activity of T cells and promote tumor cell growth. VIP receptor antagonists can inhibit this signaling, thereby preventing the reduction in anti-tumor activity of T cells and countering the immune-suppressive tumor microenvironment in PDAC (Ravindranathan et al., 2022). These antagonists can be used in combination with immune checkpoint inhibitors to enhance their therapeutic effects by mitigating the impact of the immune-suppressive tumor microenvironment (Zhang et al., 2022). Experimental studies have shown that VIP receptor antagonists alone can downregulate the expression levels of PD-1 and PD-L1 on immune cells (Li et al., 2016). Furthermore, the combination of VIP receptor antagonists with anti-PD-1 treatment significantly enhances the induction of tumor-specific T cell responses and provides protective immunity against tumor re-attack (Ravindranathan et al., 2022). Administration of anti-PD-1 alone leads to upregulation of CXCR4 expression in T cells, but this effect can be counteracted by co-administration of a VIP receptor antagonist with anti-PD-1 (Ravindranathan et al., 2022). Combination therapy has proven to be more effective than monotherapy. Although VIP receptor antagonists have shown efficacy in treating various types of tumors, their specific applications and characteristics are still under investigation and have not yet been formally implemented in clinical treatment (Moody et al., 1993; Zia et al., 1996; Ravindranathan et al., 2022).

### 5.2 Targeted therapy for CD74 and its conjugates

CD74 acts as a co-factor for MHCII molecules, and its expression levels progressively increase with PDAC progression. CD74 can also increase the secretion of GDNF through the PI_3_K/AKT/EGR-1 pathway (Zhang et al., 2021). Studies have shown a positive correlation between CD74 levels and GDNF. Since GDNF promotes the occurrence and development of pancreatic neuroplasticity invasion, targeting CD74 can lower down GDNF levels and inhibit PNI (Cavel et al., 2012). Milatuzumab, a novel immunotherapeutic drug targeting CD74, has shown promising results in clinical trials for cancer treatment (Kaufman et al., 2013). However, CD74 targeted therapy is more effective in hematological tumors compared to solid tumors (Burton et al., 2004; Govindan et al., 2013). Studies suggest that the efficacy of CD74 targeting in solid tumors can be improved through drug conjugation. For example, the Milatuzumab-SN-38 conjugate has increased the survival period of mice with solid tumor xenografts and enhanced targeting and anti-tumor toxicity (Govindan et al., 2013). Nonetheless, the specific therapeutic effects and characteristics of Milatuzumab conjugates are still under investigation and have not been widely used in clinical treatment.

Currently, therapeutic approaches that target the neuroimmune dialogue in PDAC are still in the clinical research stage, with imperfect techniques and outcomes. However, treatment methods based on the neuroimmune dialogue in PDAC show promising prospects and can be considered as future directions for PDAC treatment research.

## 6 Conclusion and future perspectives

Numerous studies have indicated that the interaction between the nervous system and immune system plays a crucial role in the development of PDAC, in addition to their individual contributions. The pancreas harbors a significant number of nerves, which not only contribute to pain through neurogenic inflammation but also engage in crosstalk with tumor cells, promoting tumor growth through perineural invasion. Moreover, the nervous system also participates in immune regulation in PDAC. While existing research has highlighted the involvement of both sympathetic and parasympathetic nerves in pro- and anti-tumor immunity, their precise roles require further investigation. Additionally, various neuromodulator factors influence the neuro-immune crosstalk together with impact in the progression of PDAC, offering potential targets for treatment. Conversely, macrophages in the immune system contribute to perineural invasion and neural remodeling by interacting with the nervous system. Circulating monocytes infiltrate the tumor’s periphery microenvironment and differentiate into macrophages in response to diverse factors, influencing tumor neural infiltration and remodeling through direct and indirect mechanisms.

Despite these advancements, several unresolved questions persist within the neuro-immune crosstalk network. It remains unknown whether additional neuromodulators are involved in this process, and if other immune cells besides macrophages communicate with the nervous system. Therapies targeting neuro-immune crosstalk are still in the preclinical stage, requiring additional research to establish their efficacy. Overall, understanding the significance of neuro-immune crosstalk in PDAC is crucial for unraveling its pathogenesis and developing novel treatment approaches.
